# A sustainable multi-task HPLC–UV method for simultaneous analysis of top neuromodulating agents in diverse pharmaceutical formulations

**DOI:** 10.1038/s41598-025-07502-8

**Published:** 2025-07-02

**Authors:** Sara El-Hanboushy, Hoda M. Marzouk, Nada S. Ayish

**Affiliations:** 1 Pharmaceutical Chemistry Department, Faculty of Pharmacy, Future University in Egypt, Cairo, 11835 Egypt; 2 Pharmaceutical Analytical Chemistry Department, Faculty of Pharmacy, Cairo University, Kasr El-Aini street, Cairo, 11562 Egypt

**Keywords:** Multi-analyte analysis, HPLC–UV, Gabapentin, Piracetam, Levetiracetam, Content uniformity testing, Dissolution testing, Assessment of environmental impact, Analytical chemistry, Sustainability

## Abstract

Epilepsy is a chronic neurological disease that affects the brain and causes a recurrent seizure. This condition affects a lot of people worldwide making it a global neurological disorder. The first line for managing epilepsy, is the use of Anti-epileptic drugs. Herein, a reliable and ecological chromatographic method was developed, verified and validated for the simultaneous determination of three approved drugs that can be co-administered in many treatment protocols for epilepsy management; Piracetam (PIR), Gabapentin (GBP) and Levetiracetam (LEV). The developed method was founded on high performance liquid chromatography (HPLC–UV). Separation was accomplished using a 5.0 μm particle size, 250 × 4.6 mm Inertsil ODS-3 C_18_ column with the UV detection set at 210.0 nm. An isocratic elution system was employed, consisting of a mixture of methanol and water in a ratio of 15:85 (v/v) at ambient temperature. The developed method demonstrated linearity throughout a range of concentration of 30.0–1000.0 µg/mL for GBP and 10.0–100.0 µg/mL for LEV and PIR. The suggested method was optimized and validated following the guidelines stated by the ICH and was utilized for the determination of the aforementioned drugs in their respective pharmaceutical formulation. Moreover, the supremacy of the developed method was further extended for monitoring the in-vitro release profile of the stated drugs and content uniformity of their marketed pharmaceutical formulations. Finally, the ecological impact of the suggested method was monitored, evaluated and compared to the official HPLC ones utilizing newly introduced greenness, blueness and whiteness assessment methods tools.

**Supplementary Information:**

The online version contains supplementary material available at 10.1038/s41598-025-07502-8.

## Introduction

Epilepsy is a brain disorder that can affect individuals of all ages and races worldwide. It is characterized by decreased level of neuronal inhibition in the cerebral cortex as a result of low levels of Gamma-aminobutyric acid (GABA), which is one of the most significant neurotransmitters in the central nervous system that has a major role in reduction of neuronal excitability through inhibition of nerve transmission^1^, cells become depolarized which trigger seizures. A persistent propensity to have seizures and the neurobiological, cognitive and social ramifications of seizures recurrence are the hallmarks of epilepsy^2^. Anti-epileptic drugs (AEDs) are the anchor of treatment. Their main goal is to achieve complete seizure eradication, reduce morbidity, mortality and improve quality of life^3^. Among the most commonly known GABA analogue drugs used in the management of epilepsy and associated seizures either solely or in-combination; Piracetam (PIR), Gabapentin (GBP) and Levetiracetam (LEV).

**Fig. 1:**
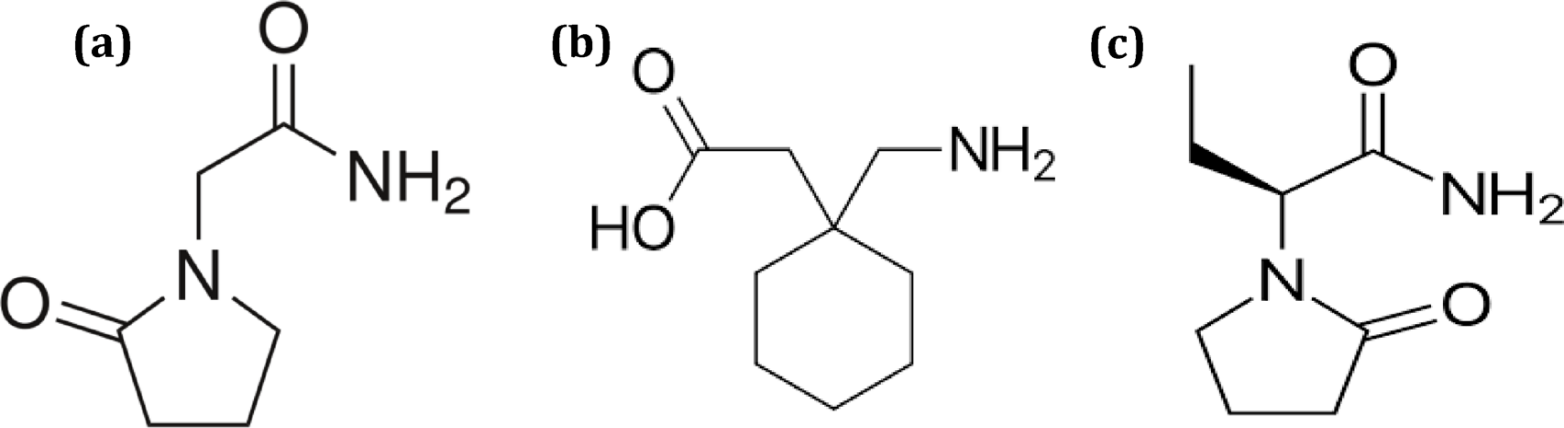
Chemical structures of (**a**) Piracetam (PIR), (**b**) Gabapentin (GBP) and (**c**) Levetiracetam (LEV).

The first drug, Piracetam (PIR) Fig. 1a, is a nootropic medication used primary in the management of some age-related cognitive disturbances including Alzheimer disease, post-stroke deficits^4^. Although piracetam show no anti-convulsant activity, it was proven that the usage of piracetam as an adjuvant therapy with anti-epileptic drugs reduces seizures severity and enhance the anticonvulsant activity of conventional anti-epileptic drugs^4^. The second drug, Gabapentin (GBP) Fig. 1b, which is an anti-epileptic medication with strong anticonvulsant properties, originally designed as a gamma-aminobutyric acid-mimetic compound for the treatment of spasticity. Gabapentin appears to block the excitatory neurotransmitters in the presynaptic area involved in epileptogensis by exhibiting strong affinity to binding sites in the brain that correspond to voltage-gated calcium channels, particularly α-2-δ-1^5^. Levetiracetam (LEV) Fig. 1c, is indicated for the management of partial onset seizure, myoclonic seizure and for generalized tonic–clonic seizures^6^. It works by attaching to the brain’s synaptic vesicle protein SV2A and modulating the release of synaptic neurotransmitters^6^. Taking into consideration that GBP combination with either PIR or LEV has proven synergistic effect in controlling seizures as stated in literature^4,7,8^. The purpose of the current work was to entrench a versatile, accurate, eco-friendly and economical chromatographic method for the simultaneous determination of multi-analyte, PIR, GBP and LEV, in a single run, making this method effective for their determination in any future pharmaceutical combination, especially that, upon searching for published work, no method was reported for the simultaneous determination of the three aforementioned analytes together. Only few reports were described for the estimation of LEV together with GBP and other anti-epileptic drugs including; LC–MS methods^9,10^ as well as LEV with either PIR and brivaracetam or with lamotrigine including HPLC methods^11,12^. Additionally, the authors tried to extrapolate the advantage of the developed method for in-vitro dissolution monitoring of the cited drugs in their respective pharmaceutical formulation. As it is known, One of the main quality attributes stated by FDA for monitoring development of pharmaceutical formulations is dissolution testing, especially for oral solid dosage form to control drug product quality, stability and batch-to-batch consistency, as it provides a simulated prediction of drug in-vivo behaviour^13^. In-vitro drug release monitoring is regularly performed in quality control unites to detect the influence of critical manufacturing variables on the final drug product. After following the dissolution conditions and parameters stated for each drug, in-vitro dissolution testing is mainly done by sampling at predetermined intervals subsequently, off-line estimation of drug released percent at each time interval using different quantification techniques^14,15^. In order for the quantification method to give a reliable result, it must be precise, repeatable and selective, capable of distinguishing between different drugs that might be co-formulated together. Making the developed method capable of monitoring the in-vitro release profile of the stated drugs.

Recently, a lot of effort endeavoured to achieve sustainability three pillars including: saving of the environment, improving quality of life and economic enhancements^16^. For this reason, in Pharmaceutical industries, the majority of scientists started to adhere to 1990’s emerging green analytical chemistry paradigm (GAC)^17^, to fulfil the sustainable development goals while protecting the environment^18–25^. In this consensus, different assessment tools were established to evaluate the environmental impact of the developed methods^26–28^. Hence, the authors adopted different assessment tools to evaluate the ecological impact of the developed method including; AGREE, blueness and whiteness assessment tools.

## Experimental

### Reagents and materials

Methanol, HPLC-grade, was acquired from Sigma-Aldrich, (Darmstadt-Germany), additionally, double distilled water was received from “Aquatron” (Staffordshire, England). Pure PIR standard, with certified purity of 99.80%, was supplied from Jiangsu GuoTai International Group, (Shanghai-China), while pure GBP standard, certified to contain 98.80% was obtained from Maps Laboratories Private Limited, (Gujarat-India). Pure LEV was provided from Hetero Labs Limited, (Hyderabad-India) with certified purity of 100.72%.

Nootropil® Tablet, claimed to contain 800.0 mg PIR, manufactured by UCB Pharma for GlaxoSmithKline, UK (B.N211202). Neurontin® Hard Gelatine Capsules, claimed to contain 400.0 mg GBP, manufactured by Pfizer PFE Switzerland (B.N. T70376) and Keppra® Film-Coated Tablets, claimed to contain 500.0 mg LEV, manufactured by UCB Pharma, Belgium (B.N: 382125). All samples were purchased from local market.

### Instrumentation and separation conditions

Separation was achieved using a liquid chromatograph system featuring: a Shimadzu pump (Model LC-20AI, Japan) with a variable wavelength ultra-violet detector (Model SPD—20 A, Japan) and 20 µL injection loop. LabSolutions software (Release 1.22 SP1) was utilized for Data acquisition and manipulation. For the separation of the three cited drugs, an Inertsil C_18_ analytical column of 250 × 4.6 mm and with a particle size (5.0 μm) was used as a stationary phase and was thermostatically controlled at 25 °C. For the mobile phase, a solution comprising methanol and water in the ratio 15:85, v/v was pumped with a flow rate of 1.5 mL/min. and detection wavelength was adjusted at 210.0 nm.

### Standard solutions

In three distinct 100-mL volumetric flasks, an amount of analytical standards equivalent to 20.0 mg of each of LEV and PIR and 200.0 mg of GBP, were separately weighed and dissolved in 50-mL water and sonicated for 10 min then the volume was completed to the final volume utilizing water which serves as a solvent to prepare a stock standard solution of 200.0 µg/mL of each of LEV and PIR and 2000.0 µg/mL of GBP.

### Procedures

#### Calibration curve creation

Discrete aliquots from the stock solutions of each drug were individually placed in three groups of volumetric flasks (each 10-mL). Using mobile phase as a solvent, the volume was adjusted to the mark to cover the concentration ranges of 10.0–100.0 µg/mL for LEV and PIR and 30.0–1000.0 µg/mL for GBP. Following the previously specified chromatographic conditions, 20.0 µL of the previously prepared solutions was injected into the chromatograph in triplicate. For calibration curve construction, a plot of the average peak areas obtained against its correlating concentration of each drug and then regression equations were calculated. Furthermore, the parameters for system suitability were computed in order to guarantee the suitability of the chromatographic system.

#### Assay of pharmaceutical formulations

Ten dosage units of each drug were separately weighed and uniformly grinded into powder. An amount equivalent to one dosage unit of each drug was weighed and transferred into three separate 100-mL measuring flasks, each contain 50-mL distilled water and sonicate for 15 min. The volume was then adjusted to the mark using water. After that, the prepared solution was filtered on 0.45 µm membrane filter to attain a sample solutions of concentration 8.0 mg/mL, 4.0 mg/mL and 5.0 mg/mL of PIR, GBP and LEV, respectively. Then, one mL of each of the prepared solutions was transferred into three separate 100-mL volumetric flasks utilizing the mobile phase mixture as a solvent to obtain a concentration of 80.0 µg/mL, 40.0 µg/mL and 50.0 µg/mL of PIR, GBP and LEV, respectively. Then, following the above-mentioned chromatographic conditions, 20.0 µL of each solution was injected into the chromatograph and the corresponding concentration was computed using the respective regression equation of each drug.

#### Dissolution testing

First of all, appropriate dilutions from each drug stock standard solutions were prepared discretely in a series of 10-mL measuring flasks using dissolution media as a solvent to simulate real samples and assure results validity. After that, calibration curves were constructed and each drug regression parameters were computed.

The dissolution of the studied drugs was monitored using USP Apparatus II (paddle) in 0.06 N HCl or water as per official monograph (GBP and LEV)^29^ or manufacturer’s method (PIR) in addition to three typical bio-relevant dissolution media, namely, 0.1 N HCl, acetate buffer pH 4.5, phosphate buffer pH 6.8^30^. The media were prepared in accordance with USP guidelines under “Buffer Solutions” section ^29^. The dissolution vessel was filled with 900-mL of medium (providing sink conditions based on studied drugs’ solubility), adjusted at 50 rpm and maintained at 37 °C. Then, the same procedures were followed for in-vitro dissolution testing of the three cited drugs where one tablet/capsule was immersed in each vessel, samples were withdrawn at previously set time intervals (10, 15, 20, 30 min), filtered, and compensated with equivalent amounts of fresh medium. The final solutions were then introduced into the chromatographic system following the separation conditions stated above. Peak areas were recorded at different dissolution media and sampling intervals. Drug concentrations were estimated using the corresponding regression equations. After that, percentage dissolution (% dissolution) were computed, plotted versus sampling time, and dissolution profiles were constructed.

#### Content uniformity testing

Ten tablets/capsules of each drug were solely tested using the proposed method to check the uniformity of the content of each formulation following the guidelines stated by the United States Pharmacopeia (USP)^31^. Each tablet/capsule was individually prepared as mentioned in the assay of pharmaceutical formulation. Proper aliquots were then withdrawn and transferred into 10-mL volumetric flasks. The volume was then adjusted to the mark utilizing mobile phase mixture. Finally, the previously mentioned procedures were followed to acquire the cited drugs concentration.

## Results and discussion

Recently, developing an analytical method capable of simultaneous quantification of multi-analyte that are structurally or pharmacologically related at a time is one of a beneficial analytical trends according to GAC concepts^32–34^. Such multi-task method is both time and energy saving and therefore more economic compared to many pharmaceutical analytical methods of analysis that is routinely used in quality control units, making multi-analyte analysis a chief goal of various analytical laboratories in the regulatory, industrial and academic environments throughout the world. Consequently, the primary goal of the current work was to create a simple, sensitive and eco-friendly method that is able to estimate multi-analyte at a single run in addition to testing different quality attributes, including dissolution testing and content uniformity of the final dosage form, to certify the quality and efficacy of the final pharmaceutical formulation.

### Optimization of separation conditions

For best performance and efficacy of the developed method, several studies were carried out to reach the optimum separation conditions of the stated drugs together in single run. Using an Inertsil C_18_ analytical column of (250 × 4.6 mm, 5.0 μm) thermostatically controlled at 25 °C as a stationary phase and adjusting detection wavelength at 210.0 nm, a fixed concentration of the stated drugs (100 µg/mL each) was used and one parameter was changed at a time in order to reach the optimum separation conditions. Different mobile phases were tried with varied composition and ratio of organic modifier. Due to the hazardous properties of most of the organic solvents (like acetonitrile) and their detrimental effect on the environment, only methanol and ethanol were tried as organic modifier. Although ethanol is widely recognized as one of the most environmentally benign organic solvents, preliminary trials demonstrated its inadequacy in achieving the required selectivity and peak sharpness for this analytical application. In contrast, methanol offered superior chromatographic performance, providing enhanced resolution, reduced analysis time, and improved system suitability parameters. From an Environmental, Health, and Safety (EHS) standpoint, methanol and ethanol exhibit comparable profiles^35–37^ as shown in Fig. S1, rendering methanol an equally viable and sustainable option. Moreover, methanol remains preferable to acetonitrile due to its lower toxicity and reduced disposal concerns. Furthermore, deionized water, phosphate buffer at different values were attempted in different ratios as aqueous phase. It was observed that upon using phosphate buffer (pH 2.8 ± 0.2), a deteriorated peak shape was observed with poor peak resolution. Upon using phosphate buffer (pH 5.0 ± 0.2), an enhanced peak shape was observed with good peak resolution but with increased retention time. After that, deionized water was attempted as an aqueous phase in different ratios. Further optimization studies confirmed that decreasing the methanol content adversely affected separation efficiency, necessitating the adoption of an optimized methanol-to-water ratio for subsequent method development.

**Fig. 2:**
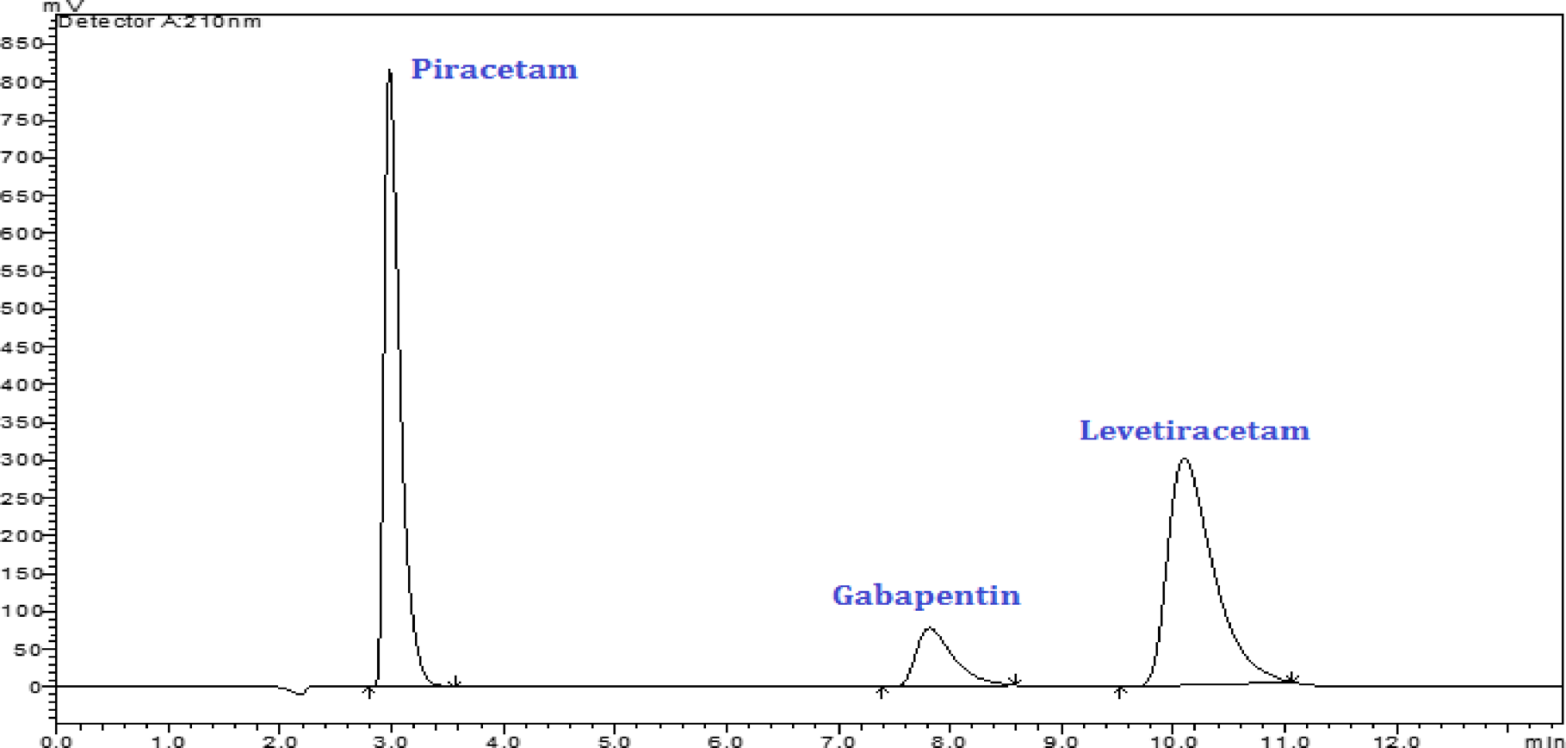
HPLC–UV chromatogram of resolved mixture of 100.0 µg/mL piracetam, gabapentin and levetiracetam under the optimized chromatographic conditions.

Optimum separation with acceptable peak shape and resolution was reached using methanol—deionized water in the ratio (15:85, v/v) in isocratic mode through 1.5 mL/min flow rate, Fig. 2. By computing the system suitability parameters Table 1, the obtained results were in great compliance with the acceptable reference ranges^38^ which ensure the efficiency of the suggested approach.

**Table 1:** System suitability parameters of the proposed HPLC–UV method.

Parameters	PIR	GBP	LEV
Retention time (*t*_*R*_)	2.976 ± 0.1	7.812 ± 0.1	10.097 ± 0.1
Capacity factor (*k*′)	0.6	3.2	4.4
Selectivity factor (*α*)^a^	5.33	1.38	--
Resolution (*R*_*s*_)^b^	11.15	3.41	--
Tailing factor (*T*)^c^	1.25	1.50	1.25
Theoretical Plate number (*N*)^d^	1600	2179	2916
Height equivalent to theoretical Plate (*HETP*) [cm]^e^	0.016	0.011	0.009

^a^α =*k*′_2_/*k*′_1_, where *k*′ is the capacity factor; *k*′ = (t_R _− t_0_)/t_0_.

^b^R_s_ = [2 (t_R2 _− t_R1_)]/(W_1_ + W_2_), Where W is peak width at 5% from the baseline of the peak height.

^c^T = W_0.05_/2f, where W_0.05_ is the width of the peak at 5% height and f is the distance from peak maximum to the leading edge of peak.

^d^N = 16 (t_R_/W)^2^, where W is the peak width.

^e^HETP = L/N, where L is the column length (cm).

### Protocols for evaluating sustainability and greenness of the suggested method

The compliance of the presented HPLC method to the hypotheses of the green and white analytical chemistry was listed throughout different state-of-the-art assessment tools, namely, Analytical GREEnness Metric Software (AGREE) for green tracking check, Blue Applicability Grade Index (BAGI) software which address the practicality of the method and the RGB-12 model for the comprehensive evaluation of ecological, sustainability and functionality aspects. In this study, implementation of such up-to-date tools ensure a comprehensive and multi-dimensional evaluation of the proposed analytical method. Each tool independently assesses specific aspect of sustainability, and their combined use provides a broader and more robust perspective by addressing different dimensions of environmental impact.

The adherence of the suggested method with GAC concepts was comprehensively compared in parallel to the official methods^39,40^ as follows:

#### GREEnness analytical tool (AGREE)

In 2020, Pena-Pereira developed the novel AGREE software, a printed tool for evaluating greenness^28,41^. It operates via an easy to use downloaded calculator (v.0.5) that can be obtained at https://mostwiedzy.pl/AGREE. The AGREE metric combines the twelve items of green analytical chemistry (GAC). The automatically created pictogram is detached into twelve sections, each of which has a unique colour spectrum extending from dark green (= 1) to dark red (= 0). The chiefly score, expressed as a fraction of unity between zero and one, is displayed in the centre of the pictogram, with a higher value representing better sustainability. The colour combination that results in colour in the centre is the 12 AGREE pictogram segments’ output. When utilising the colour dark green, the optimal green method receives a score of one.

**Fig. 3:**
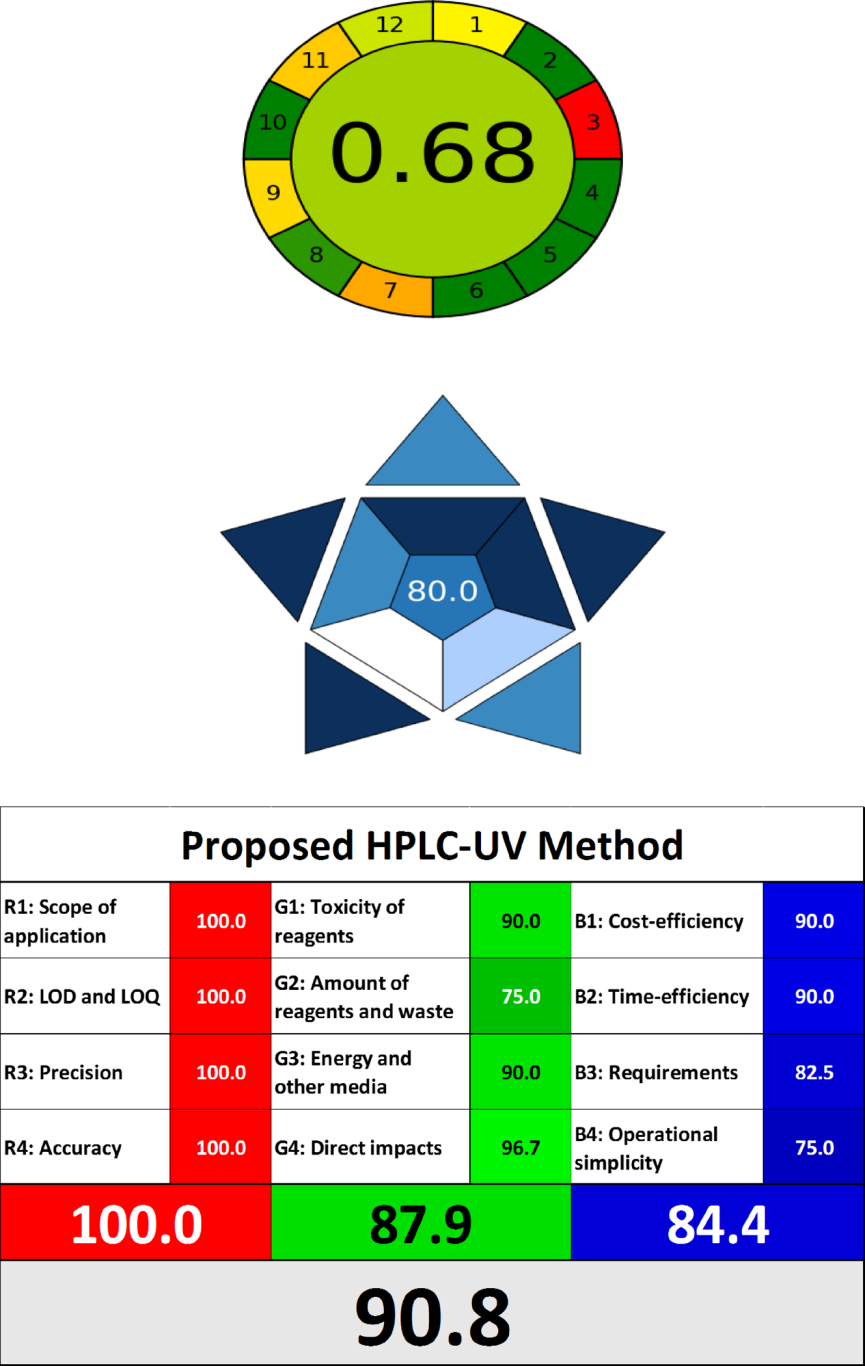
Greenness, blueness and whiteness evaluation of the proposed HPLC–UV method via AGREE, BAGI and RGB12 assessment tools.

As shown in Fig. 3 and Fig. S2, the developed HPLC methodology captures greenness with the highest overall score (0.68) with darker green color in the centre compared with the official methods of the studied drugs.

#### Blue applicability grade index (BAGI)

The BAGI tool is one new metric for assessing the applicability of an analytical procedure. In addition to the well-settled green metrics, BAGI is largely focused on the practical main points of White Analytical Chemistry^42^. Blue Applicability Grade Index (BAGI) is a novel metric developed in 2023, introducing a comprehensive approach to assessing the practical aspects of WAC. It is accessible through an open-source and user-friendly application (v0-9–2) (mostwiedzy.pl/bagi). Unlike conventional green metrics that focus on environmental influence, BAGI precisely evaluates ten essential applicability attributes related to the analytical procedure and analysed sample. The yielded result is presented as a visual graphical asteroid pictogram, as well as a numerical score relating to it at the center. This pictogram is composed of ten sections colored with multiple shades of blue to indicate differing levels of compliance (white for non-compliance, light blue for low, blue for moderate, and dark blue for high). The ultimate BAGI score, ranges from 25 for the approach with the worst applicable characteristics to 100 for the method with the best behaviour. A detailed review of several areas of the BAGI findings revealed superior BAGI score (80) indicated for the suggested HPLC representing favorable features of the “BLUE” criteria and excellent applicability, as shown in Fig. 3, relative to the official HPLC methods of the proposed drugs (75), Fig. S2.

#### White analytical chemistry (WAC) assessment

The RGB 12 model was updated via Nowak et al.^43^. The White Analytical Chemistry (WAC) method integrates the principles of sustainable development into analytical chemistry by balancing research validity with environmental preservation^44,45^. It evaluates analytical methods across three complementary components—analytical effectiveness (red), environmental impact (green), and economic and practical utility (blue)—each represented by four specific criteria. The “white” result reflects a holistic, balanced, and sustainable analytical approach. *Red Band (Analytical Effectiveness)*: The proposed HPLC–UV method demonstrated superior analytical performance with low LOD and LOQ values (R2), ensuring high sensitivity. Its broad application scope (R1) makes it suitable for diverse application benchmarks, while trueness (R3) and precision (R4) meet ICH guidelines, confirming the method’s reliability and reproducibility. These results affirm that the method excels in analytical effectiveness, achieving a perfect red score of 100%. *Green Band (Environmental Impact)*: The HPLC–UV method demonstrated strong alignment with green chemistry principles: *Reagent Toxicity (G1)*: The use of biodegradable methanol and water minimizes environmental harm. *Reagent and Waste Consumption (G2)*: The method requires small volumes of solvents, reducing chemical waste. *Energy Efficiency (G3)*: With an analytical runtime of 10 min, the method minimizes energy use compared to longer procedures. *Impact on Living Beings (G4)*: The study involved no animal testing or genetically modified organisms, ensuring ethical compliance. Overall, the method achieved a green score of 87.9%. *Blue Band (Economic and Practical Utility)*: The proposed method is cost-efficient (B1) due to its reliance on affordable reagents and standard equipment. Its short run time (B2) improves throughput, while its operational simplicity (B4) minimizes training and infrastructure requirements. Additionally, the method eliminates the need for extensive transportation or specialized equipment, reducing overall costs and logistical complexity. The resulting blue score of 84.4% indicates high sustainability and practicality, with slight room for improvement in time and cost efficiency. The proposed HPLC–UV method achieved the highest whiteness score of 90.8%, significantly outperforming the official methods for each drug, as shown in Fig. 3 and Fig. S2, which demonstrates its potential as a sustainable alternative for multi-drug analysis. To streamline the presentation of results, Table S1 summarizes the key scores across the different assessment tools.

While the application of modern assessment tools such as AGREE, BAGI, and RGB-12 provides valuable insights into the sustainability of the proposed analytical method, it is important to acknowledge their limitations and biases: *AGREE Tool Limitations*: the AGREE tool is designed to summarize the compliance of an analytical method with the 12 principles of green analytical chemistry using a weighted scoring system. Nevertheless, there isn’t a definite guideline for establishing the significance of each sector within the 12 sections. Furthermore, it overlooks the procedures for sample preparation during the assessment. Considering *BAGI Tool Limitations*: BAGI focuses on specific practical aspects of the analytical process, such as reagent use and toxicity, but does not provide a full life cycle perspective. Additionally, as BAGI provides a single aggregated score, it may obscure details about individual parameters, making it less granular compared to other tools like WAC. These tools are best suited for comparative assessments rather than absolute evaluations of environmental impact. As such, their results should be interpreted with an awareness of their distinct features. Future studies could further enhance greenness assessments by incorporating broader life cycle analysis methodologies to complement these tools and ensure a more complete evaluation of sustainability.

Although the multi-analyte procedures can be challenging in terms of method development and the need for more strict system control. The successfulness of the presented method lies on its capability to reduce the utilization and consumption of the used solvent and instrumental energy. In addition, offering a more straightforward, transparent approach that enhances reproducibility and reduces errors. The proposed method’s high throughput capability, particularly in pharmaceutical dissolution studies and dosage form analysis, allows for the analysis of multiple analytes per hour, increasing overall efficiency without compromising accuracy. These attributes make it superior, aligning with green chemistry principles and offering a more sustainable, efficient solution for modern pharmaceutical analysis as compared to those for the official methods.

### Method validation

In accordance with ICH criterion, the suggested HPLC–UV procedure was validated^46^.

#### Linearity

Linearity of the proposed method was examined through calibration curves plotting connecting the measured peak areas to the respective concentrations of every drug and regression parameters were then computed. Good correlation coefficients (≥ 0.999) were obtained spanning the 30.0–1000.0 µg/mL concentration range for GBP and 10.0–100.0 µg/mL for LEV and PIR, Table 2. Moreover, the validity of the developed method was demonstrated by evaluating the precision and trueness at the limit of quantification (LOQ), with acceptable results.

**Table 2:** Method validation parameters for determination of piracetam, gabapentin and levetiracetam by the proposed HPLC–UV method.

Parameter	PIR	GBP	LEV
Linearity range (µg/mL)	10.0–100.0	30.0–1000.0	10.0–100.0
Slope	79,381.95	1795.53	85,380.11
Intercept	323,642.01	− 11,742.52	79,017.58
Correlation coefficient (*r*)	0.9995	0.9997	0.9997
Accuracy (mean ± RSD%)	99.85 ± 0.53	99.03 ± 0.64	100.11 ± 0.70
Precision (RSD%)			
Repeatability^a^	0.60	0.45	0.54
Intermediate precision^b^	0.89	0.62	0.65
Robustness^c^ (RSD%)			
Changing wavelength (± 1 nm)	0.24	0.55	0.78
Changing mobile phase ratio (± 1%)	0.31	0.66	0.50
Changing flow rate (± 0.1mL/min)	0.79	1.47	0.89

^a^Intra-day precision [average of three different concentration of three replicates each (n = 9) within the same day].

^b^Inter-day precision [average of three different concentration of three replicates each (n = 9) repeated on three successive days].

^c^Average of three determinations.

#### Precision

Three different concentraion levels of each component were examined in triplicates, following the opitimized conditions, within the same day as well as on three sequential days. Table 2 displays the acquired results which show acceptable method precision as indicated by good %RSD (< 2%).

#### Trueness

In order to evaluate the trueness of the developed approach, a minimum of six distinct pure samples of every component spanning the specified concentration range were analysed over three times. The respective concentration of individually sample was obtained using the associated regression equation of individually component. Acceptable recoveries percent were attained as indicated in Table 2, where % Recovery of 98–102% is typical for an assay of an active ingredient in a drug product.

#### Robustness

To assure the suggested method dependability, small deliberate changes were made to the conditions of separation, such as changing the scanning wavelength (± 1.0 nm), mobile phase ratio (± 1%) and flow rate (± 0.1 mL/min), followed by calculating the %RSD to monitor method performance. As shown in Table 2, the acceptable value of the obtained %RSD (< 2%), indicates the robustness of the suggested method with sufficient resolution (Fig. S3).

### Assay of pharmaceutical formulation

Eventually, the optimised approach was utilised and verified for the identification of the medications mentioned. Each in its respective pharmaceutical formulation, Table 3. Finally, furthermore, implementation of the technique of standard addition was conducted to verify the validity of the proposed method and positive outcomes were attained as illustrated in Table 3.

**Table 3:** Quantitative estimation of piracetam, gabapentin and levetiracetam in respective dosage form and application of standard addition technique.

Dosage form	Nootropil ® Tablet (BN: 211,202)	Neurontin ® Capsules (BN: T70376)	Keppra® Tablet (BN: 382,125)
	PIR	GBP	LEV
Found% a ± RSD%	100.89 ± 0.22	98.89 ± 0.59	99.04 ± 0.43
Standard addition technique			
%Recovery of the pure added ± SD^a^	101.68 ± 0.30	99.15 ± 0.39	100.57 ± 1.06

^a^Average of three determinations.

**Table 4:** Results of content uniformity testing for determining piracetam, gabapentin and levetiracetam in the studied pharmaceutical dosage forms using the proposed HPLC–UV method.

Unit no	Label claim (%)		
	PIR	GBP	LEV
1	103.96	96.48	94.77
2	106.42	96.57	98.65
3	105.59	96.66	97.16
4	103.98	99.36	94.85
5	108.46	99.70	98.35
6	106.86	99.28	97.97
7	104.01	94.94	94.63
8	108.41	95.17	98.67
9	108.45	95.30	97.95
10	104.02	95.17	95.47
Mean	106.01	96.86	96.85
SD	1.97	1.89	1.72
RSD%	1.86	1.95	1.77
Acceptance value (AV)*	0.218	6.176	5.778
Maxi. allowed AV (L1)	15	15	15

*Acceptance value = 2.4 × SD with maximum allowed level (L1) is 15.

### Dissolution monitoring

**Fig. 4:**
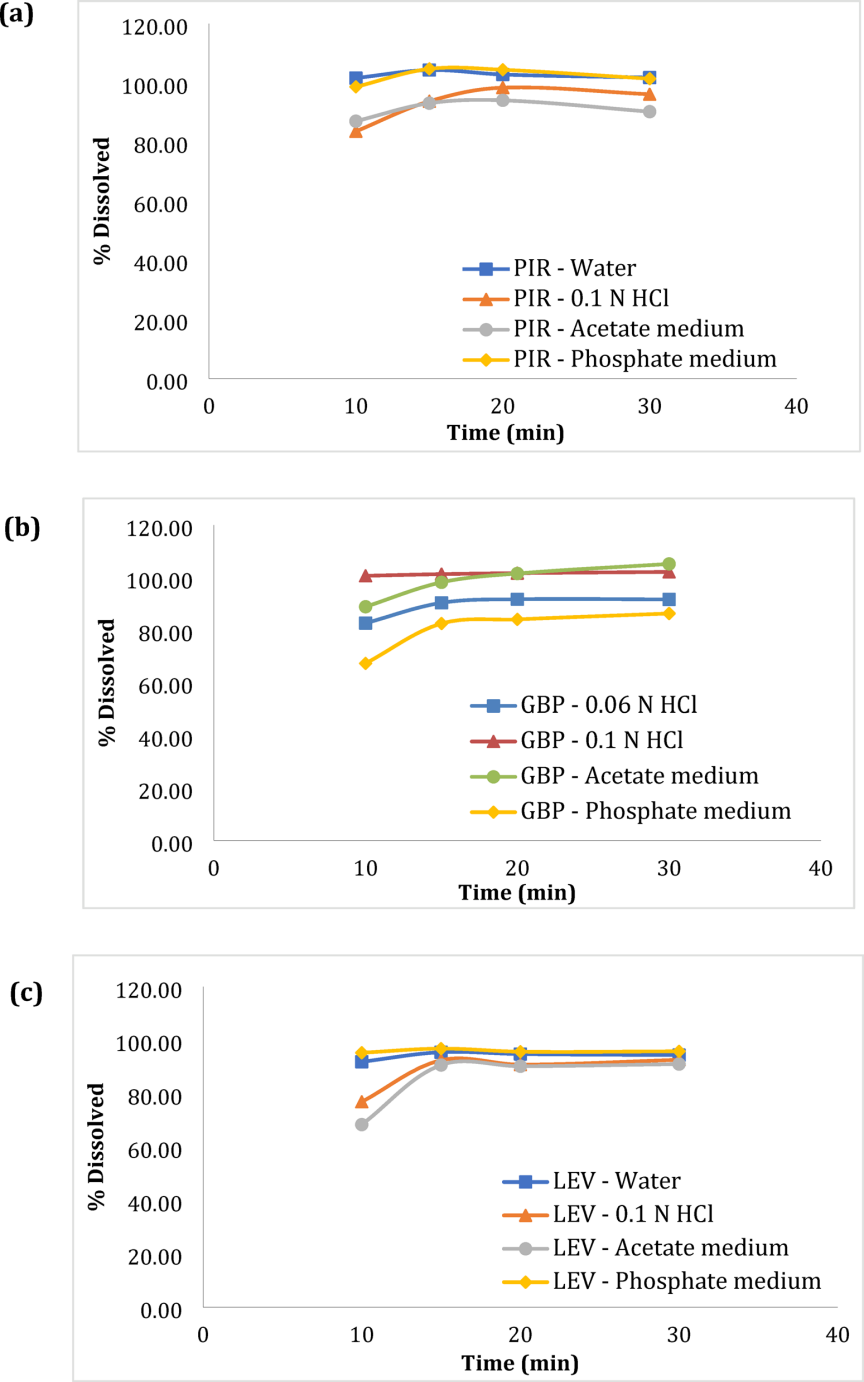
Dissolution profiles of (**a**) piracetam, (**b**) gabapentin and (**c**) levetiracetam in Nootropil® Tablets, Neurontin® Capsules, Keppra® Tablets, respectively, in various dissolution media using the developed HPLC–UV method.

United State Pharmacopoeia (USP) states that the exemption of active pharmaceutical ingredient (API) from the designed drug product is what mostly determines drug absorption following oral drug delivery^29^. Thereby, to augur the drug products’ in-vivo performance, in-vitro dissolution monitoring is performed. Using the suggested approach, the USP parameters were followed for monitoring the dissolution of each drug product as mentioned above. Then constructing the dissolution profile that links time to the % dissolved. Figure 4 demonstrates that the obtained results were in close approximate with the approved USP limits certifying the validity of the recommended approach for dissolution observing to the stated drug’s pharmaceutical formulations.

### Content uniformity testing

Regarding single dose preparations, it is crucial that the patient gets the exact dose of medication that is stated on the label. That’s why content uniformity testing is considered one of the most important tests that is conducted routinely in quality control units^47,48^. Acceptance criteria given by Rules’ USP that stipulate that the calculated acceptance value (AV) should be below 15, as this is the highest allowable limit. (L1). Acceptance value (AV) was computed utilizing the equation below:

$${\text{AV}} = M - \overline{X} + K \times SD$$

where, M referred to the reference value, $\overline{X }$ referred to the mean of samples recoveries, K referred to the acceptability constant, and finally SD referred to standard deviation of samples.

According to the USP^31^, if the sample size is ten and $\overline{X }$ lies within the range of (98.5%-101.5%), the reference value (M) shall equal $\overline{X }$ and K will be fixed at 2.4. As demonstrated in Table 4**.** The calculated AV was found less than the highest permissible level of acceptance (L1) which proofs the uniformity of dosage forms content.

## Conclusion

A novel and reliable chromatographic methodology was developed for the simultaneous determination of three neuromodulating agents that can be used merely or co-administrated in different treatment protocol for various neurological disorders. The developed method is simple, cost-effective and displays acceptable performance over a wide concentration range. Additionally, the proposed method proved to be able to estimate the studied drugs in raw materials and marketed pharmaceutical formulations. Moreover, the method was effectively implemented to monitor the in-vitro dissolution profiles of the cited drugs in addition to testing the content uniformity of dosage unites. Thus, the proposed method has the potential to separate, quantify and analyse the aforementioned medication in any future combined pharmaceutical formulation. Finally, the ecological impact of the developed method was assessed utilizing the most recently developed assessment metric tools and were found to outstand over the official HPLC procedures for each drug. Hence, the developed method is considered an optimum alternative for various applications in quality control laboratories.

## Electronic supplementary material

Below is the link to the electronic supplementary material.

Supplementary Material 1

## Data Availability

All data generated or analysed during this study are included in this published article.

## References

[1] Kuffler, S. W. & Edwards, C. Mechanism of gamma aminobutyric acid (GABA) action and its relation to synaptic inhibition. *J. Neurophysiol.* **21**, 589–610 (1958). 13599049 10.1152/jn.1958.21.6.589

[2] Beghi, E. The epidemiology of epilepsy. *Neuroepidemiology* **54**, 185–191 (2020). 31852003 10.1159/000503831

[3] Sander, J. W. The use of antiepileptic drugs—Principles and practice. *Epilepsia* **45**, 28–34 (2004). 15315513 10.1111/j.0013-9580.2004.455005.x

[4] Fischer, W., Kittner, H., Regenthal, R., Russo, E. & De Sarro, G. Effects of piracetam alone and in combination with antiepileptic drugs in rodent seizure models. *J. Neural Transm.* **111**, 1121–1139 (2004). 15338329 10.1007/s00702-004-0155-6

[5] Gupta, Y. Gabapentin as an effective treatment for epilepsy. *J. Neurol. Neurophysiol.* **14**, 1–4 (2023).

[6] Abou-Khalil, B. Levetiracetam in the treatment of epilepsy. *Neuropsychiatr. Dis. Treat.* **4**, 507–523 (2008). 18830435 10.2147/ndt.s2937 PMC2526377

[7] Lu, X.-C.M. et al. Combination therapy of levetiracetam and gabapentin against nonconvulsive seizures induced by penetrating traumatic brain injury. *J. Trauma Acute Care Surg.* **83**, S25-34 (2017). 28452872 10.1097/TA.0000000000001470

[8] Wlaz, A. et al. Synergistic interaction of levetiracetam with gabapentin in the mouse 6 Hz psychomotor seizure model—A type II isobolographic analysis. *Curr. Issues Pharm. Med. Sci.* **28**, 204–207 (2015).

[9] Juenke, J. M., Brown, P. I., Johnson-Davis, K. L. & McMillin, G. A. Simultaneous quantification of levetiracetam and gabapentin in plasma by ultra-pressure liquid chromatography coupled with tandem mass spectrometry detection. *Ther. Drug Monit.* **33**, 209–213 (2011). 21297550 10.1097/FTD.0b013e31820b1fce

[10] Palte, M. J. et al. Development and validation of an ultra-performance liquid chromatography–Tandem mass spectrometry method for the concurrent measurement of gabapentin, lamotrigine, levetiracetam, monohydroxy derivative of oxcarbazepine, and zonisamide concentrations in Se. *Therapeutic Drug Monit.* **40**, (2018). 10.1097/FTD.0000000000000516 PMC6501579 29994986

[11] Mansour, N. M., El-Sherbiny, D. T., Ibrahim, F. A. & El Subbagh, H. I. Validation of a specific reversed-phase HPLC method for the quantification of three racetams; Piracetam, levetiracetam, and brivaracetam in the presence of Co-administered drugs in their pharmaceuticals; greenness assessment and application to biological. *Microchem. J.* **181**, 107703 (2022).

[12] Ulusoy, S., Ulusoy, H. İ, Locatelli, M. & Kabir, A. Titania-based fabric phase sorptive extraction approach for the determination of antiepileptic drugs, levetiracetam and lamotrigine in urine samples using high-performance liquid chromatography-photo diode array detection. *J. Chromatogr. A* **1719**, 464737 (2024). 38387152 10.1016/j.chroma.2024.464737

[13] Shah, V. P. Progressive applications of dissolution, its impact and implication in the pharmaceutical world. *J. Pharm. Sci.* **102**, 2895–2897 (2013). 23728620 10.1002/jps.23608

[14] Marzouk, H. M., Ayish, N. S., El-Zeany, B. A. & Fayed, A. S. Eco-friendly chromatographic platforms for simultaneous determination and impurity profiling of an antihypertensive ternary pharmaceutical mixture. *Sustain. Chem. Pharm.* **32**, 100978 (2023).

[15] Kamel, M. A., Nessim, C. K., Michael, A. M., Abbas, S. S. & Marzouk, H. M. A sustainable HPLC method coupled with diode array detection for versatile quantification of telmisartan, chlorthalidone and amlodipine in a fixed-dose antihypertensive formulation and dissolution studies. *BMC Chem.* **18**, 166 (2024). 39267180 10.1186/s13065-024-01276-2 PMC11391801

[16] Purvis, B., Mao, Y. & Robinson, D. Three pillars of sustainability: In search of conceptual origins. *Sustain. Sci.* **14**, 681–695 (2019).

[17] Anastas, P. T. Green chemistry and the role of analytical methodology development. *Crit. Rev. Anal. Chem.* **29**, 167–175 (1999).

[18] Marzouk, H. M., Ayish, N. S., El-Zeany, B. A. & Fayed, A. S. An eco-friendly separation-based framework for quantitative determination and purity testing of an antihypertensive ternary pharmaceutical formulation. *BMC Chem.* **17**, 1–13 (2023). 36899384 10.1186/s13065-023-00926-1 PMC10007836

[19] Fayed, A. S., Ayish, N. S., El-Zeany, B. A. & Marzouk, H. M. Novel dalfampridine-selective green potentiometric membrane sensors for in-line dissolution profiling of its extended release tablets. *Microchem. J.* **165**, 106127 (2021).

[20] Ali, S. N., Saad, S. S., Fayed, A. S. & Marzouk, H. M. Intelligent spectrophotometric resolution platforms for the challenging spectra of ipratropium and fenoterol in their combination inhaler with ecological friendliness assessment. *Sci. Rep.* **14**, 22406 (2024). 39333660 10.1038/s41598-024-72431-x PMC11436838

[21] Marzouk, H. M., Gouda, A. S., Rezk, M. R. & Abdel-Megied, A. M. Innovative eco-friendly stability-indicating HPLC-PDA method for simultaneous determination of the emerging antiviral drugs against COVID-19 infection molnupiravir and favipiravir; degradation kinetic studies along with LC-MS based structure elucidation. *Microchem. J.* **205**, 111197 (2024).

[22] El-Hanboushy, S., Marzouk, H. M., Fayez, Y. M., Abdelkawy, M. & Lotfy, H. M. Eco-friendly spectrophotometric evaluation of triple-combination therapies in the treatment strategy of patients suffering from hypertension during coronavirus pandemic—Spectralprint recognition study. *Spectrochim. ActaPart A Mol. Biomol. Spectrosc.* **280**, 121523 (2022). 10.1016/j.saa.2022.121523 PMC9212944 35759933

[23] El-Hanboushy, S. et al. Design of green polypyrrole-based solid-contact ion-selective sensors for determination of antihypertensive drugs in combined dosage forms and spiked human plasma. *J. Electrochem. Soc.* **170**, 37520 (2023).

[24] Lotfy, H. M., El-Hanboushy, S., Fayez, Y. M., Abdelkawy, M. & Marzouk, H. M. Computational intelligence spectrophotometric scenarios for screening and quantification of single-dose triple therapy banned by the world anti-doping agency in some sports. *Microchem. J.* **196**, 109581 (2024).

[25] Gamal, S., Mandour, A. A., Mohamed, G. G., Salih, S. A. & Ahmed, D. A. Simultaneous spectrophotometric determination of recombined sofosbuvir, ledipasvir and paracetamol together as commonly repurposed drugs for COVID-19 treatment. *Future J. Pharm. Sci.* **9**, 71 (2023).

[26] Mansour, F. R., Bedair, A. & Locatelli, M. Click analytical chemistry Index as a novel concept and framework, supported with open source software to assess analytical methods. *Adv. Sample Prep.* **14**, 100164 (2025).

[27] Mansour, F. R., Płotka-Wasylka, J. & Locatelli, M. Modified GAPI (MoGAPI) tool and software for the assessment of method greenness: Case studies and applications. *Analytica* **5**, 451–457 (2024).

[28] Locatelli, M. et al. Green profile tools: Current status and future perspectives. *Adv. Sample Prep.* **6**, 100068 (2023).

[29] *United States Pharmacopeia/National Formulary (USP 30-NF 25), United States Pharmacopeial Convention.* (2007).

[30] Shah, V. P. et al. FDA guidance for industry 1 dissolution testing of immediate release solid oral dosage forms. *Dissolut. Technol.* **4**, 15–22 (1997).

[31] U.S. Pharmacopoeial Convention. *(905) Uniformity of Dosage Units. Stage 6 Harmonization.* vol. 3 (2011).

[32] Elbordiny, H. S., Elonsy, S. M., Daabees, H. G. & Belal, T. S. Development of a sustainable multianalyte MEKC method for quantitation of the antihyperlipidemic drugs ezetimibe together with three statins. Greenness and whiteness appraisal studies. *BMC Chem.* **17**, 1–13 (2023). 37742031 10.1186/s13065-023-01040-y PMC10518094

[33] Vincent, U., Serano, F. & von Holst, C. Validation of a multi-analyte HPLC method for the determination of carotenoids used as feed additives in fish and poultry feed: Results of an interlaboratory study. *Food Addit. Contam. Part A Chem. Anal. Control Expos. Risk Assess.* **38**, 396–408 (2021). 10.1080/19440049.2020.1869325 33481680

[34] Dos Santos, B. P. et al. A multi-analyte LC-MS/MS method for the determination of 57 pharmaceuticals and illicit drugs in plasma, and its application to poisoning cases. *J. Pharm. Biomed. Anal.* **222**, 115082 (2023). 36183577 10.1016/j.jpba.2022.115082

[35] Capello, C., Fischer, U. & Hungerbühler, K. What is a green solvent? A comprehensive framework for the environmental assessment of solvents. *Green Chem.* **9**, 927–934 (2007).

[36] Saleh, A. M., Saleh, O. A., Hassan, R. Y. A., Badawey, A. M. & Marzouk, H. M. A novel quality-by-design assisted HPLC-DAD method for the simultaneous quantification of tryptophan, tryptophol, and voriconazole for early diagnosis and prognosis of fungal infections decoding quorum sensing phenomenon. *J. Chromatogr. B Anal. Technol. Biomed. Life Sci.* **1257**, 124571 (2025). 10.1016/j.jchromb.2025.124571 40186998

[37] Alder, C. M. et al. Updating and further expanding GSK’s solvent sustainability guide. *Green Chem.* **18**, 3879–3890 (2016).

[38] *Reviewer Guidance, Validation of chromatographic methods*. (Center for Drug Evaluation and Research (CDER), 1994).

[39] United States Pharmacopeia/National Formulary. USP-NF, Rockville, MD: United States Pharmacopeia. (2024).

[40] *European Pharmacopoeia 11.0: Published in Accordance with the Convention on the Elaboration of a European Pharmacopoeia*. (European Directorate for the Quality of Medicines \& Healthcare, 2022).

[41] Pena-Pereira, F., Wojnowski, W. & Tobiszewski, M. AGREE—Analytical GREEnness metric approach and software. *Anal. Chem.* **92**, 10076–10082 (2020). 32538619 10.1021/acs.analchem.0c01887 PMC7588019

[42] Manousi, N., Wojnowski, W., Płotka-Wasylka, J. & Samanidou, V. Blue applicability grade index (BAGI) and software: A new tool for the evaluation of method practicality. *Green Chem.* **25**, 7598–7604 (2023).

[43] Nowak, P. M., Wietecha-Posłuszny, R. & Pawliszyn, J. White analytical chemistry: An approach to reconcile the principles of green analytical chemistry and functionality. *TrAC Trends Anal. Chem.* **138**, 116223 (2021).

[44] El-Hanboushy, S., Marzouk, H. M., Fayez, Y. M., Abdelkawy, M. & Lotfy, H. M. Sustainable spectrophotometric determination of antihypertensive medicines reducing COVID-19 risk via paired wavelength data processing technique—Assessment of purity, greenness and whiteness. *Sustain. Chem. Pharm.* **29**, 100806 (2022). 35992213 10.1016/j.scp.2022.100806 PMC9376343

[45] Marzouk, H. M. et al. Sustainable chromatographic quantitation of multi-antihypertensive medications: Application on diverse combinations containing hydrochlorothiazide along with LC–MS/MS profiling of potential impurities: Greenness and whiteness evaluation. *BMC Chem.* **17**, 1–17 (2023). 37598182 10.1186/s13065-023-01015-z PMC10439576

[46] *ICH harmonised tripatite guidline validation of analytical procedures: Text and methodology Q2(R1) Guideline on validation of analytical procedures: Methodology developed to complement the parent guideline* (2005).

[47] Ali, S. N., Saad, S. S., Fayed, A. S. & Marzouk, H. M. Chromatographic fingerprinting of ipratropium and fenoterol in their novel co-formulated inhaler treating major respiratory disorders; application to delivered dose uniformity testing along with greenness and whiteness assessment. *BMC Chem.* **18**, 157 (2024). 39192312 10.1186/s13065-024-01265-5 PMC11350986

[48] Marzouk, H. M., Ayish, N. S., El-zeany, B. A. & Fayed, A. S. Green spectrophotometric resolution platforms of spectrally overlapping signals of antihypertensive ternary pharmaceutical formulation: Application to content uniformity testing. *J. Anal. Pharm. Res.* **78**, 1244–1254 (2023).

