# Supplementary material for: A sustainable multi-task HPLC–UV method for simultaneous analysis of top neuromodulating agents in diverse pharmaceutical formulations
Source: Sci Rep. 2025 Jul 2;15:22724. doi: 10.1038/s41598-025-07502-8 (PMC12215692; doi:10.1038/s41598-025-07502-8)
Supplement: Supplementary file 1 — Supplementary Material 1 [file 41598_2025_7502_MOESM1_ESM.docx]

Supplementary material

**For**

**A sustainable multi-task HPLC-UV method for simultaneous analysis of top neuromodulating agents in diverse pharmaceutical formulations**

*Sara El-Hanboushy ^1^, Hoda M. Marzouk ^2*^, Nada S. Ayish ^2^*

^1^ Pharmaceutical Chemistry Department, Faculty of Pharmacy, Future University in Egypt, 11835, Cairo, Egypt

^2^ Pharmaceutical Analytical Chemistry Department, Faculty of Pharmacy, Cairo University, Kasr El-Aini street, 11562, Cairo, Egypt.

**
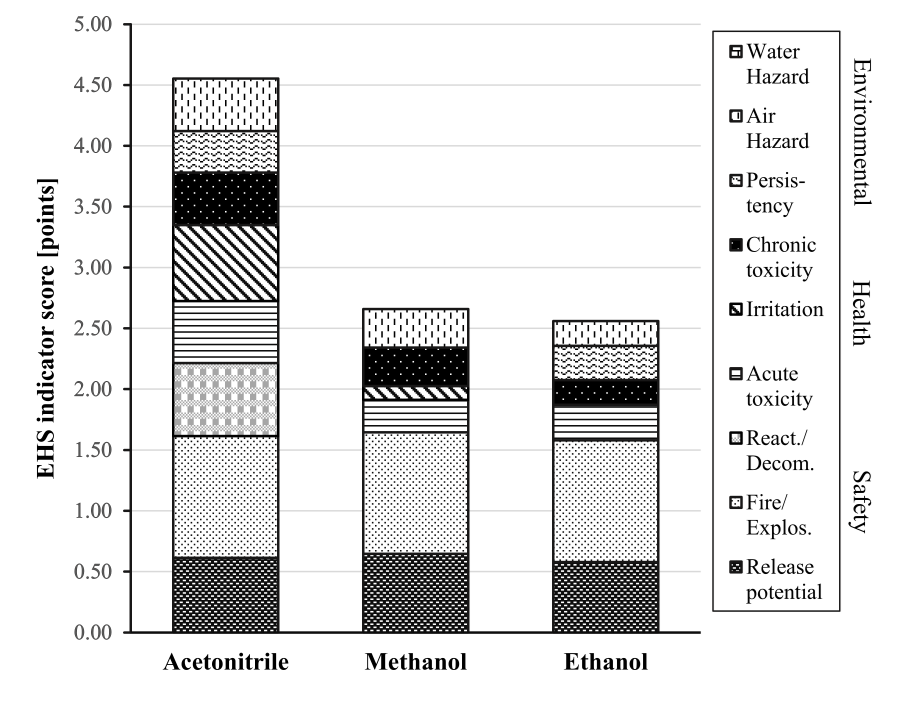
**

**Fig. S1.** EHS assessment for commonly used solvents in chromatographic methods, illustrating the relatively similar EHS score of methanol and ethanol.

**(a)**

**(b)**

**(c)**


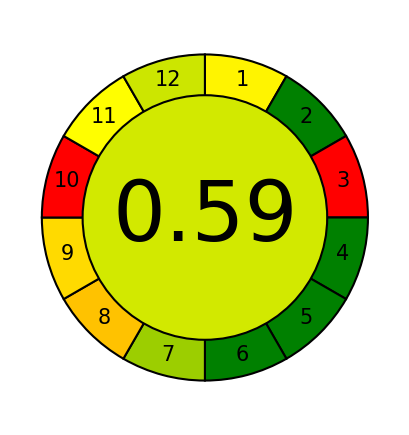

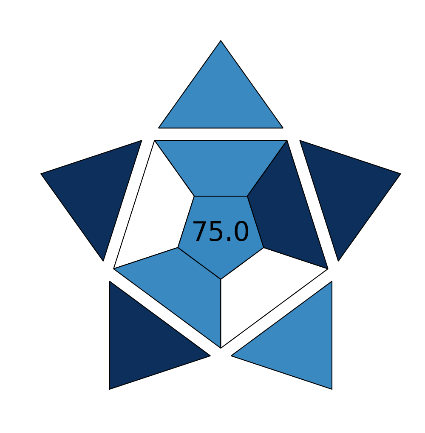

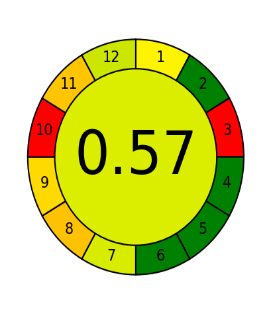

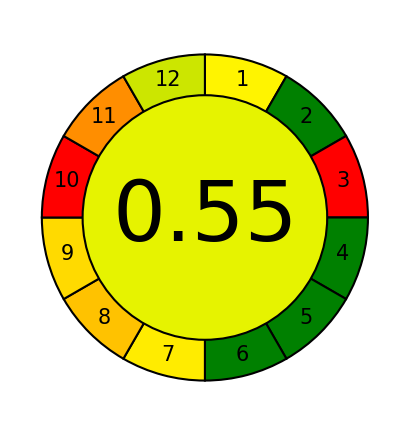

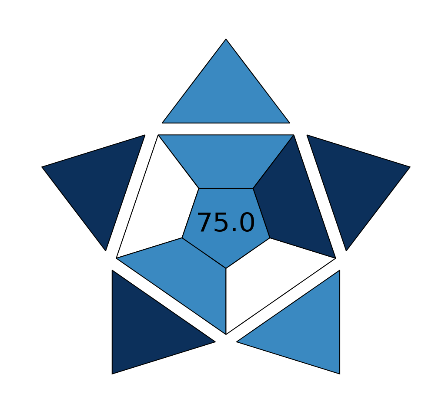

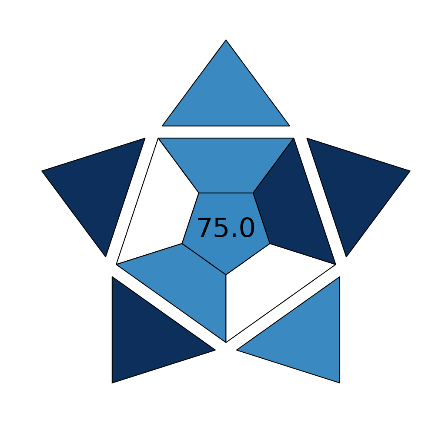

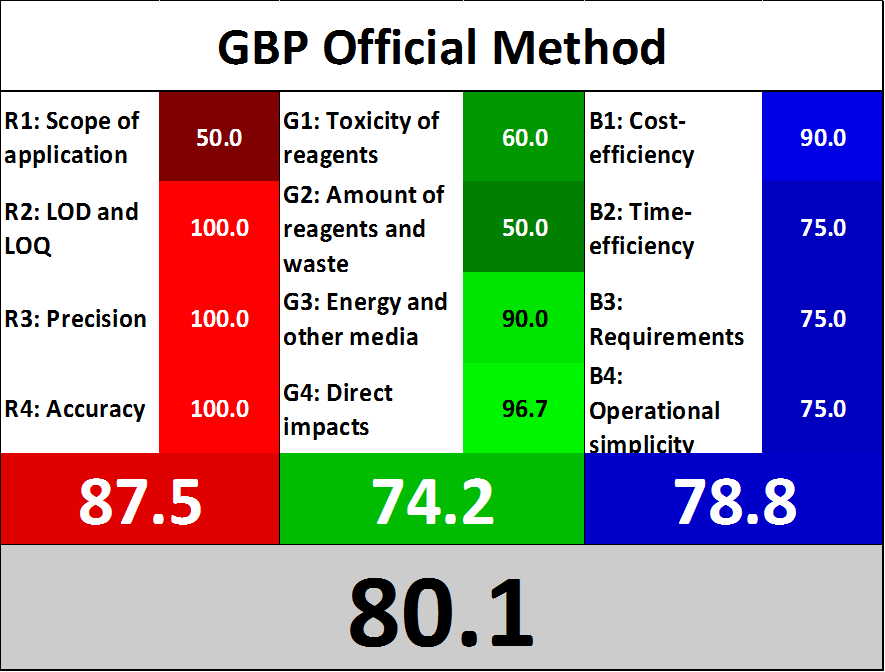

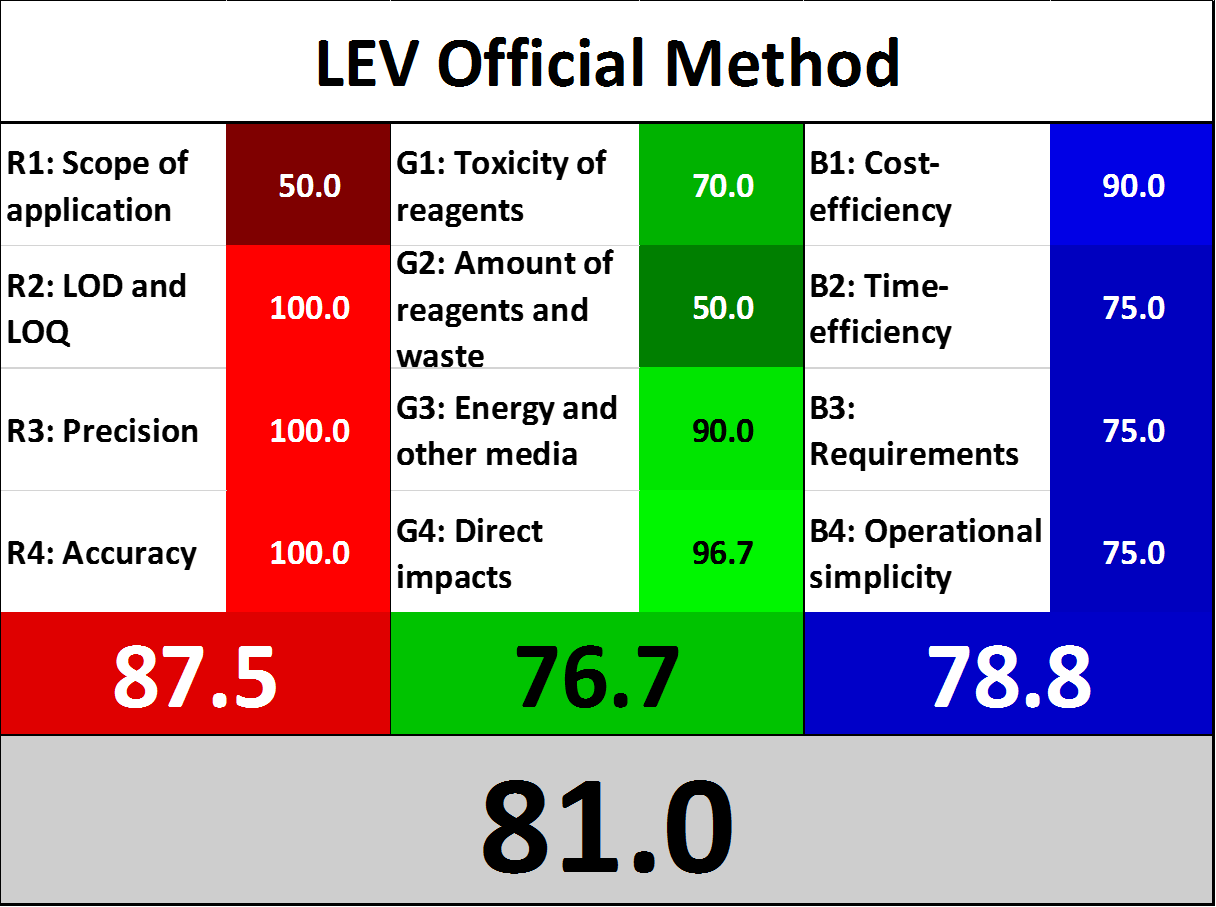


**Fig. S2.** Greenness, blueness and whiteness assessment of the official HPLC methods for (a): PIR **[40]**, (b): GBP **[39]** and (c): LEV **[39],** *via* AGREE, BAGI and RGB12 tools.

**
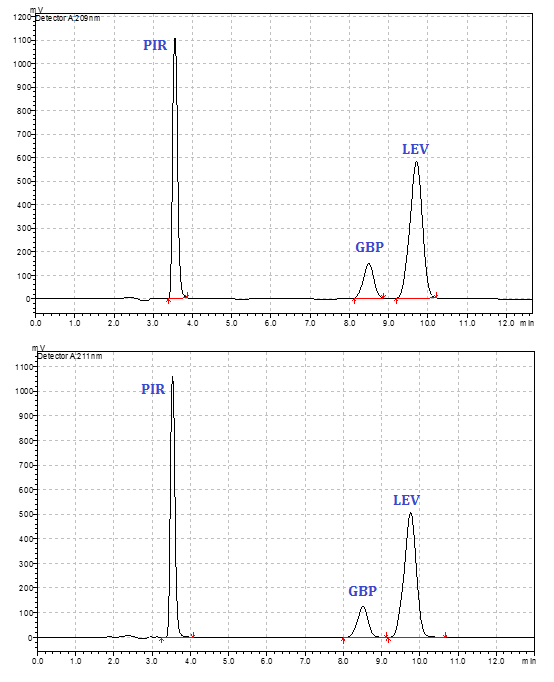
**

**Fig. S3.** HPLC-UV chromatograms of a resolved mixture of PIR, GBP and LEV upon changing the scanning wavelength (± 1.0 nm).

**Table S1.** Comparison of evaluation scores for the proposed HPLC-UV method and official methods using the greenness (AGREE), blueness (BAGI), and whiteness (RGB12) assessment tools.

| **Tool/Metric** | **Proposed HPLC-UV Method** | **Official Methods** | | |
| --- | --- | --- | --- | --- |
|  |  | **PIR [41]** | **GBP [40]** | **LEV [40]** |
| **AGREE Score** | 0.68 | 0.59 | 0.57 | 0.55 |
| **BAGI Score** | 80.0 | 75.0 | 75.0 | 75.0 |
| **WAC Red Band (R%)** | 100.0 | 87.5 | 87.5 | 87.5 |
| **WAC Green Band (G%)** | 87.9 | 79.2 | 74.2 | 76.7 |
| **WAC Blue Band (B%)** | 84.4 | 78.8 | 78.8 | 78.8 |
| **Overall WAC Score (%)** | 90.8 | 81.8 | 80.1 | 81.0 |
